# Do memory concerns and self-efficacy predict academic performance in undergraduate students with memorization practice?

**DOI:** 10.3389/fpsyg.2025.1515533

**Published:** 2025-08-14

**Authors:** Maura Pilotti, Dana Alzahid, Nouf Faisal, Arifi Waked, Omar J. El-Moussa

**Affiliations:** ^1^Department of Sciences and Human Studies, Prince Mohammad bin Fahd University, Khobar, Saudi Arabia; ^2^Department of Management and Information Systems, Prince Mohammad bin Fahd University, Khobar, Saudi Arabia; ^3^Cognitive Science Research Center, Prince Mohammad bin Fahd University, Khobar, Saudi Arabia; ^4^Department of Student Affairs, Prince Mohammad bin Fahd University, Khobar, Saudi Arabia

**Keywords:** prospective memory, retrospective memory, academic self-efficacy, Middle East, subjective memory

## Abstract

**Introduction:**

The main aim of the present study was to examine the extent to which self-reported prospective and retrospective memory concerns and academic self-efficacy predict academic attainment in sophomore and junior undergraduate students.

**Method:**

Subjective memory and self-efficacy reports were completed by a convenience sample of an understudied population of bilingual Saudi Arabian female students with a verbatim memory educational past. They were enrolled in a self-assessment course.

**Results:**

Responses illustrated greater prospective than retrospective memory concerns. A modest relationship between memory concerns and academic self-efficacy was also found, suggesting that memory issues play a minor role in the confidence students possess in their academic abilities. Academic attainment was minimally related to academic self-efficacy and not at all to self-reported prospective or retrospective memory difficulties.

**Discussion:**

These findings illustrate that awareness of memory failures does not correspond to an inability to satisfy academic demands, since it may bring about the use of compensatory strategies. Implications for teaching and learning are discussed.

## Introduction

The life of undergraduate students is cognitively demanding. As such, it is not surprising that it can deplete the cognitive resources necessary to remember things that have been accomplished (retrospective memory) and those that need to be accomplished (prospective memory). In educational settings, these two forms of memory emerge as separate but not equal entities. Retrospective memory is deemed a key component of undergraduate students' performance. Indeed, remembering what has been studied is an ordinary demand placed on students by all the courses in which they are enrolled (Unsworth et al., 2013). Prospective memory is also an important but less obvious component of performance. It involves remembering intentions developed in the past that need to be realized in the present. Intentions that qualify as prospective memory events are those that are not active in working memory until the moment they are to be executed. For instance, students may forget about an upcoming quiz and go out with friends instead of studying. As a result, they may fail the quiz the next day. Alternatively, students may forget to meet with tutors or advisors, thereby jeopardizing their chances to clarify class materials while, at the same time, appearing negligent (Basso et al., 2023; Moeller et al., 2021). The hallmark of prospective memory is that attention is focused on something other than the intention to be recollected, while an ongoing activity must be temporarily stopped for the intention to be executed. A prospective memory becomes a realized intention when students understand the meaning of the memory cue that brings the record of the intention into awareness. The memory cue capable of activating the intention can be either an environmental stimulus (e.g., a note on a calendar or a beep from a cellphone) or an internal stimulus (e.g., a thought suddenly coming to mind; Anderson et al., 2017). Furthermore, the intention may engage working memory (e.g., at the end of an interesting class meeting, planning to ask the instructor for an appointment) or long-term memory (e.g., planning to check the schedule of an advisor the next day on campus). Thus, prospective and retrospective memory tasks differ slightly. A retrospective memory task involves the encoding of an event, a varying retention delay, and then a test as to whether the past event is retained. Instead, a prospective memory task involves the encoding of an intention, a varying retention delay, and then a test of whether the intention is executed (McBride and Workman, 2017).

Both retrospective memory and prospective memory have been reported to be positively associated with processing speed, working memory, and measures of crystallized and fluid intelligence (Uttl et al., 2018). Forgetting in prospective memory has been primarily attributed to failures of attentional monitoring (Anderson et al., 2018; for an alternative account, see Heathcote et al., 2015), whereas forgetting in retrospective memory has been attributed to a variety of sources, encompassing encoding and retrieval processes (e.g., working memory capacity and attentional control). Evidence also exists that the neural correlates of failures of these two types of memory are different (West and Krompinger, 2005). They are also different in their social consequences. Students' retrospective memory failures tend to be interpreted by others as memory difficulties, whereas prospective memory failures are interpreted as character flaws (Basso et al., 2023; Moeller et al., 2021).

Subjective memory is defined as how individuals view their memory functioning, including memory strategies, past processing outcomes, and beliefs about the strengths and weaknesses of their memory abilities (Csábi et al., 2025). Thus, it is not surprising that the examination of subjective memory is regarded as providing insights into students' cognitive functioning, confidence in their abilities (i.e., self-efficacy), and ultimately performance (Pearman, 2009; Unsworth et al., 2012). Students' subjective memory, which reflects how they view performance in prospective and retrospective tasks, engages their metacognition (i.e., awareness and control of their cognitive abilities). Metamemory specifically refers to metacognition about memory processes and outcomes. It can be considered a fundamental aspect of students' self-regulation of learning and thus potentially a contributor to academic performance (Balashov et al., 2018; Kancharla et al., 2023). It entails students' self-evaluation of their likely memory performance based on similar past experiences (e.g., knowing that appointments may be forgotten), the particular encoding circumstances of the event to be remembered (e.g., having been given choices about the time of the appointment), as well as behavioral adjustments in response to the evaluation (e.g., creating a physical record of the appointment by entering its time and day into the calendar to avoid memory lapses; Cauvin et al., 2019). Metamemory reports are found to be relatively accurate predictors of actual memory performance (Kliegel and Jäger, 2006; Melo et al., 2021). Interestingly, the few studies that have investigated students' views of both types of metamemory have yielded conflicting results. For instance, (Melo et al. 2021) found that Brazilian university students reported more prospective than retrospective memory difficulties, mostly concerning short-term functioning. (Zare et al. 2014), instead, found no significant difference in the reports of Iranian university students. Equally unclear is the relative contribution of prospective and retrospective metamemory to academic performance at the university level.

## The study

The research questions and hypotheses tested in our study pertain to an understudied population of female students with a past emphasizing verbatim learning. Saudi Arabia offers a sample of this population. Indeed, before entering undergraduate studies, young adults complete their primary and secondary education under a pedagogy that emphasizes rote rehearsal (Alrashidi and Phan, 2015). The latter generally comprises memorization (i.e., the encoding and storing of information in long-term memory) and recitation (i.e., the reactivation of information stored in long-term memory to preserve it). In this cultural context, rote rehearsal is not viewed as a mindless activity but as an opportunity to further reflect and understand relevant information (Boyle, 2006; Yusuf, 2010). Namely, the more information is repeated, the more time students have to think about its meaning. As a result, repetition is seen as increasing the opportunities not only for developing durable memory records but also for engaging in elaborative rehearsal (Al-Ghazali, 2011; Boyle, 2006; Günther, 2006).

Practicing a skill generally improves performance outcomes contingent on that skill (Ericsson, 2003; Ericsson et al., 1980; Higbee, 1997; Kliegl et al., 1987). However, (Schnitzspahn et al. 2011) found that memory practice led to underconfidence with retrospective memory reports but not with prospective memory reports, whereas (West and Mulligan 2019) found evidence of underconfidence for both. Among the student population selected for our study, (El Alaoui et al. 2019) reported that false recall rates (a measure of retrospective memory failures) were lower than those of published norms gathered from participants without such practice. Although correct recall rates were not significantly different, false recall rates declined as the breadth of students' self-reported recitation practice increased. (Pilotti et al. 2022) also found that self-reported moderate practice with memorization and recitation (i.e., verbatim memory practice) predicted undergraduate students' grade point average (GPA). Thus, at the very minimum, it is reasonable to assume that in this population, failures of either retrospective or prospective memory may be particularly noticeable.

In the extant literature, a key aspect of academic performance is the drive to “get things done” even in the face of obstacles. The confidence students possess in their abilities to “get things done” is generally referred to as self-efficacy. According to (Bandura 2001), it is a key building block of human agency, and thus a propeller of human activities, including those related to learning. For instance, learners with high self-efficacy respond to challenging situations by enhancing effort and displaying persistence, whereas learners with low self-efficacy tend to avoid such situations (Alhadabi and Karpinski, 2020). Learners with high self-efficacy tend to credit poor outcomes to events that can be controlled (e.g., effort), whereas learners with low self-efficacy tend to credit such outcomes to personal inadequacies (Stajkovic and Sommer, 2000). Self-efficacy beliefs are often portrayed as enhancing learners' commitment, effort, and perseverance, as well as reducing negative emotions during learning (e.g., anxiety; Travis et al., 2020). Thus, it comes as no surprise that in the extant literature, academic self-efficacy is often positively related to performance in school (Tossavainen et al., 2021; van Zyl et al., 2022). Specifically, academic self-efficacy is the belief that one possesses the cognitive, behavioral, emotional, and social resources required to perform well on academic tasks (Nielsen et al., 2018). Academic self-efficacy is not only related to performance but also to students' engagement (Doo and Bonk, 2020). Although overall self-efficacy beliefs are often found to be moderately linked to good academic performance (Bouih et al., 2021; Honicke and Broadbent, 2016), negative or null correlations have been reported when self-efficacy fosters overconfidence, thereby leading to less effort being exerted (Al Kuhayli et al., 2019; Pilotti et al., 2021).

In Saudi Arabia, young women have been recently granted the right to pursue educational and career choices equal to those of their male counterparts. Gender stereotypes from a strict patriarchal past are difficult to shed at once, thereby hindering the academic success of these women in a variety of ways. Most noticeably, young women find themselves struggling to define their self-confidence (Al Alhareth et al., 2015). On one side, they are held in high regard as tokens of progress and expected to succeed academically and professionally. On the other side, their competence is questioned, and fear of failure is instilled, as they are seen as challenging traditional gender roles (Al-Bakr et al., 2017). Consequently, the evidence regarding the relationship between self-efficacy beliefs and academic attainment is mixed for undergraduate female students of Saudi Arabian descent (Al Kuhayli et al., 2019; Pilotti et al., 2021, 2022). Focusing on this population, we ask the following questions:

Q1 Are students' reports of prospective and retrospective memory abilities (metamemory judgments) different?

Q2 Are prospective and retrospective metamemory reports linked to academic self-efficacy beliefs?

Q3 Do prospective and retrospective metamemory reports and academic self-efficacy beliefs predict academic performance?

According to (McBride and Workman 2017), prospective memory tasks rely heavily on retrospective memory functions for successful execution. As a result, they both decline with increasing delays. Other similarities in metamemory are also reported. For instance, practice with either prospective or retrospective memory retrieval reduces students' confidence in their memory abilities (West and Mulligan, 2019). (Melo et al. 2021), however, found differences in metamemory among Brazilian university students. Specifically, they found more frequent reports of prospective short-term memory failures. Among students of Indian descent, (Khan and Sharma 2007) reported that prospective memory failures were rated as more frequent than retrospective memory failures. In agreement with (Khan and Sharma 2007), (Melo et al. 2021) found that short-term failures were also more frequent in prospective than retrospective reports.

The life of a university student may account for these differences. In such a life, retrospective memory tasks are as frequent as prospective memory tasks. However, failures may be differentially noticeable. Consider that students are routinely subjected to examinations in the many classes in which they enroll. Thus, failed attempts to retrieve information encoded in the near or distant past are regularly occurring events through which students gain awareness of their retrospective memory abilities. Namely, difficulties remembering some information during an examination may not be classified as an unusual event. Prospective memory tasks are typically related to students' management of events. Forgetting an intention generally triggers clear and strong reactions not only in the student who experiences the lapse but also in others. Think about the embarrassment of forgetting to show up for a test and its consequences concerning social perceptions and academic evaluations. Also, consider that afterward the event may be mentioned frequently, not only by the parties involved but also by unknown others as rumors spread in students' social networks.

It is important to note that when students are given a metamemory task, such as introspecting about their prospective and retrospective memory, they are asked to evaluate their recollection of past episodes of either type of memory. Namely, they are asked to perform a retrospective memory task in which the distinctiveness of a memory trace, and thus the ease of its retrieval, may enhance its likelihood of being remembered (Pilotti et al., 2009; Rawson and Van Overschelde, 2008). Prospective memory failures may be especially distinctive records in students' minds and, as a result, be reported as occurring at a higher frequency.

If distinctiveness defines students' reports of prospective and retrospective memory failures, then prospective memory lapses will be reported with a higher frequency than retrospective memory failures (H1-a). However, in the specific student population selected for our study, any memory lapse may be highly noticeable, thereby equating the metamemory reports of prospective and retrospective lapses (H1-b). Alternatively, the current availability of technological devices (e.g., cell phones with built-in alarm clocks and calendars) that aid the recall of intentions may produce another pattern of metamemory reports. Namely, prospective memory failures may be judged as less likely than retrospective memory failures (H1-c).

Besides metamemory, academic self-efficacy is also of interest. As noted earlier, the term refers to students' beliefs in their capabilities to plan and perform the necessary actions to attain desired academic outcomes (Nielsen et al., 2018). It is thought to determine how students feel, think, and behave in educational settings. In the selected population of young learners with a verbatim memory past, beliefs in one's academic success may depend on self-views of memory abilities. Indeed, if students perceive that they have memory problems, such beliefs may be accompanied by low self-efficacy and perhaps poor performance. Little evidence exists, however, as to whether prospective and retrospective metamemory reports are linked to academic self-efficacy. Thus, it is hypothesized that if students' views of their memory abilities play a relevant role in their self-confidence to get things done academically, metamemory reports of prospective and retrospective memory lapses will be inversely related to academic self-efficacy (H2-a). If lapses of prospective or retrospective memories vary in distinctiveness, links to academic self-efficacy will differ in strength (H2-b).

Ultimately, what matters is academic success as measured by performance indices. The metacognitive framework of (Nelson and Narens 1990) proposes an interplay between monitoring (i.e., the subjective evaluation of one's memory processes) and control (i.e., the cognitive and behavioral consequences of evaluation). The relationship between monitoring and control entails object-level cognitions (i.e., encoding and retrieving information) and meta-level cognitions (i.e., overseeing and updating the object-level operations). Thus, if the meta-level modifies the object level to induce optimal adaptation to environmental demands, students' memory and learning outcomes responsible for academic performance may be expected to benefit. This framework, however, does not include references to self-efficacy beliefs. As such, it offers no guidance as to whether prospective and retrospective metamemory reports and academic self-efficacy are equally effective predictors of academic performance in the selected student population. Studies have reported that both are linked to academic success. For instance, in samples of European and U.S. university students, (van Zyl et al. 2022) found that academic self-efficacy predicted self-reported performance. They argued that students' feeling of mastery over the demands of academic tasks enhances their engagement and motivation to perform such tasks (Tossavainen et al., 2021) or, more specifically, shapes how challenges are viewed (as opportunities to learn or setbacks; Richardson et al., 2012; Tossavainen et al., 2021). (Ismaiel 2017) reported that retrospective metamemory predicted achievement in second-language learners. Instead, (Wilhite 1990) found that the best predictor of course grades was university students' self-assessment of their memory abilities. Academic self-efficacy (as measured by the Self-Concept of Academic Ability Test, SCAAT, of Thomas et al., 1987) was less effective in predicting course grades. Of course, in the selected population of students with a verbatim memory past, whether metamemory reports and academic self-efficacy differentially predict academic performance is a matter to be investigated. If indeed the exercise of memory functions is seen as a particularly relevant component of academic performance by these students, metamemory reports will predict academic performance measures (i.e., GPA and course grades) more strongly than academic self-efficacy (H3-a).

## Method

### Participants

The participants were 521 female undergraduate students enrolled in a course dedicated to self-assessment. The course included students in their second (39%) or third year (61%) from all academic majors: 44% were enrolled in STEM programs (i.e., engineering, computer science, and architecture), and 56% were enrolled in non-STEM programs (i.e., business, law, and interior design). The participation rate was 89%. All students were of Saudi Arabian descent. They possessed Arabic as their first language, English as their second language, and a verbatim learning background. They were admitted to undergraduate programs after passing a standardized English competency test (IELTS) with an overall score of 6 or above. For these students, English language learning started in elementary school and continued through high school and preparatory English language programs. Their ages ranged from 18 to 32 years.

### Materials and procedure

At the start of the semester, students were given two questionnaires to fill out after having consented to participate: the Prospective and Retrospective Memory Questionnaire (PRMQ) of (Crawford et al. 2003), and the General Academic Self-Efficacy Scale (GASES) of (Nielsen et al. 2018), and (van Zyl et al. 2022). The questionnaires were preceded by a few demographic questions inquiring about their age, major, and educational background.

The PRMQ entailed 16 statements about memory failures (see Appendix 1) that participants had to rate on a 5-point Likert scale, including very often (5), quite often (4), sometimes (3), rarely (2), and never (1). Numerical scores were not visible to participants but were exclusively used for statistical analyses. Statements could be classified as pertaining to either prospective or retrospective memory. Within each type, statements could entail either self-cued memory or environmentally cued memory, as well as either short-term or long-term memory. The PRMQ contained an equal number of each type of statement. The overall minimal score was 16, and the maximum score was 80. A Cronbach's alpha of 0.88 attested to the internal consistency of the entire questionnaire.

The GASES contained 5 items that measured academic self-efficacy on a 5-point Likert scale ranging from strongly disagree (1) to strongly agree (5), with 3 as the neutral point. Items measured self-efficacy without reference to a specific discipline (i.e., “I generally manage to solve difficult academic problems if I try hard enough”). As per the other scale, numerical scores were not visible to participants but were exclusively used for statistical analyses. Cronbach's alpha was 0.79.

Along with the estimation of Cronbach's alpha reliability, test-retest reliability was estimated on a sample of 10 participants who took the questionnaires approximately a week apart. The correlation between the scores of time 1 and time 2 was 0.90 for PRMQ and 0.91 for GASES. The face validity of each questionnaire for the current student population (Nevo, 1985) was estimated by two students who independently judged each statement for the extent to which it appeared to measure what it was intended to measure. Students were asked to rate each statement on a 5-point scale: “extremely suitable”, “very suitable”, “adequate”, “inadequate”, and “irrelevant and therefore unsuitable”. All statements of the PRMQ or GASES received a score of either “extremely suitable” or “very suitable”, leading to an interrater agreement of 0.96 for PRMQ and 0.98 for GASES. Important to know that evidence of the reliability and validity of both the PRMQ and GASES has been provided by earlier research (e.g., Crawford et al., 2003; Nielsen et al., 2018).

Grades acquired during the first year were collected from the Office of the Registrar. They included holistic grades (i.e., GPA) or grades from two compulsory communication courses that had been taken by all students before enrollment in the assessment course. These courses were selected not only for their commonality but also for their having been described in students' end-of-semester course reports as taking a heavy toll on time and management skills. They were also the courses for which students across all majors received a grade. Communication course grades and GPA served as instances of the demands of the first year of college life.

During debriefings, anonymized comments made by students were collected and submitted to thematic analysis. Themes were developed by relying on the guidelines of thematic analysis (Braun and Clarke, 2021) devoted to a coding reliability approach. Coding was intended to merely summarize as much as possible the participants' remarks, which were used to understand their responses to the questionnaires. Two independent raters with expertise in behavioral science research categorized students' remarks. Their inter-rater agreement was 97%. Only agreed-upon comments were used to interpret students' responses to questionnaires.

Before participation, students were informed that the study aimed to understand college learners and thus to obtain information that could be used to improve teaching and learning at the selected university. Informed consent, which conformed to the APA ethical guidelines for the treatment of human participants, was then collected. As soon as performance records were matched with self-reports, all potentially identifying information was deleted from the data sets before performing statistical analyses. The data-collection process was approved by the Deanship of Research to comply with the guidelines of the Office for Human Research Protections of the U.S. Department of Health and Human Services (PMU2023-2024). Informed consent was obtained from all participants. Any potentially identifying information was deleted after the matching of the different data sources was carried out. Thus, no identifying information was present in the data set submitted to statistical analyses.

## Results

The descriptive statistics (mean, M, and standard deviation, SD) of students' responses and grades are displayed in Table 1. Inferential statistical analyses were organized by the research questions that our study asked. The probability of committing a Type I error was set to < 0.05.

**Table 1:** Descriptive statistics.

**Memory failure concerns (range: 1–5)**	***M***	***SD***
Prospective	2.91	0.76
Retrospective	2.68	0.71
Self-Efficacy (range: 1–5)	4.03	0.74
GPA (range: 1.66–4.00)	3.24	0.48
Course Grades (range: 59–98)	78.73	6.61

### Q1: Are students' prospective and retrospective metamemory reports different?

A One-way repeated measures ANOVA with type of memory concern (prospective or retrospective memory) was conducted on the participants' responses to the PRMQ. Skewness and Kurtosis analyses indicated that the data met the normality assumption (Byrne, 2010). In this analysis, more flaws were reported for prospective memory than retrospective memory (*F*_(1, 520)_ = 110.21, *MSE* = 0.118, *p* < 0.001, *partial eta*^2^ = 0.18]. These findings supported H1-a, which predicted that if distinctiveness defined students' metamemory reports, prospective metamemory lapses would be reported with a higher frequency than retrospective memory failures. By default, H1-b and H1-c were not supported.

### Q2: Are prospective and retrospective metamemory reports linked to academic self-efficacy?

Pearson correlation analyses were conducted on students' responses to the PRMQ and GASES to determine whether memory reports and self-efficacy views were linked. There was a negative relationship between reported memory deficiencies and academic self-efficacy, for both reports of prospective memory failures (*r* = 0.11, *n* = 521, *p* = 0.011), and retrospective memory failures (*r* = 0.15, *n* = 521, *p* < 0.05). No coefficient of determination (*CoD*; i.e., percentage of variance in a PRMQ dimension that is accounted for by academic self-efficacy), however, was greater than 3%. Thus, the contribution of self-efficacy to any metamemory dimensions was minimal at best.

These findings partially supported H2-a, which hypothesized that if students' views of memory play a relevant role in their self-confidence concerning academic matters, metamemory reports of prospective and retrospective memory lapses would be inversely related to academic self-efficacy reports. Although all relationships were inverse, the contribution of metamemory to academic self-efficacy was minor. H2-b did not receive clear support.

### Q3: Do prospective and retrospective metamemory reports and academic self-efficacy predict academic performance?

Pearson correlation analyses were conducted on students' responses to the PRMQ and GASES to determine whether memory reports and self-efficacy views predicted communication grades and GPA. Academic self-efficacy (as measured by GASES) predicted students' GPA (*r* = 0.18, *n* = 521, *p* < 0.001) and communication grades (*r* = 0.14, *n* = 521, *p* = 0.01). The coefficients of determination were minor ( ≤ 3%), suggesting that academic self-efficacy played a minor role in students' actual performance. GPA and communication grades failed to be predicted by any of the prospective and retrospective memory dimensions (*r*s ≤ 0.02, *ns*). This finding confirmed that university-level performance relies on much more than mere memory, even for students with a background in verbatim memory. Thus, a student may notice and report memory difficulties. However, the role such difficulties play in academic performance is seen as negligible. No support for H3-a was found. This hypothesis predicted that if the exercise of memory functions was seen as a relevant component of academic performance by these students, metamemory reports would predict academic performance measures (i.e., GPA and course grades) more strongly than academic self-efficacy.

## Discussion

The findings of the present investigation can be summarized into three main points. First, students noticed more memory failures for prospective than retrospective retrieval. Overall, our results are consistent with those of (Melo et al. 2021), who found that Brazilian university students reported more prospective than retrospective memory difficulties, mostly concerning short-term functioning. They are inconsistent with those of (Zare et al. 2014), who found no significant difference in the reports of Iranian university students. Interestingly, if the responses to the PRMQ's scale in earlier studies are averaged, our students' memory concerns do not appear to be notably higher. For instance, in the study conducted by (Crawford et al. 2003), the average ratings of prospective and retrospective memory concerns of students from the United Kingdom were 2.52 and 2.34, respectively. In the study conducted by (Zare et al. 2014), the ratings of prospective and retrospective memory concerns of Iranian students were 2.26 and 2.14, respectively. Notwithstanding an extended practice with memorization, the memory concerns of our students did not differ much from those of students in the United Kingdom who could be alleged to have had less experience with memorization. They also did not differ much from those of Iranian students who could be said to have been exposed to similar memorization practices.

Second, although there was an overall negative relationship pattern between academic self-efficacy and self-reported memory failures, the adverse contribution of failures to academic self-efficacy was minor at best. Third, students' performance (as measured by first-year GPA and communication grades) was minimally predicted by academic self-efficacy and not at all by memory failures. The findings regarding self-efficacy are consistent with studies that reported self-efficacy beliefs to predict good academic performance (Bouih et al., 2021; Honicke and Broadbent, 2016; van Zyl et al., 2022). The findings regarding subjective memory and performance are also in agreement with those of (Csábi et al. 2025), who reported no link between subjective memory functioning and objective cognitive performance.

The most important implication of our findings is that students' awareness of everyday memory failures might serve as an effective countermeasure. Indeed, the likely reason for students' academic performance not suffering from self-reported memory difficulties may be that students activated strategies that were intended to compensate for the occurrence of expected memory failures (Dismukes, 2012). In agreement with this proposal are the remarks made by students during debriefing sessions. Students confirmed that after noticing instances of failures in the past, they instituted guardrails to avoid reoccurrences. Cellphones were frequently cited as tools that students used to be reminded of things to do, thereby yielding useful environmentally cued prompts for prospective memory. Spaced exposure to information was cited as a technique for ensuring retrospective retrieval from long-term memory of information for tests and assignments. To avoid misplacing objects (e.g., cellphones, keys, etc.), students mentioned the need to be organized as well as the use of technology, such as Bluetooth trackers, all of which counteracted retrospective short-term memory lapses.

Students explained their reasons for the adoption of countermeasures. Dire consequences ensued from forgetting to carry out intended actions, such as taking a scheduled test (a prospective memory failure) or forgetting previously learned instructions, such as the steps to follow to apply for a scholarship (a retrospective memory failure). Yet, students tended to note more prospective than retrospective memory lapses and to view the former as more unpleasant. They acknowledged that their lives in and outside the classroom placed heavy demands on planning, task management, and sustained attention as much as on memory abilities. Concerning prospective memory, students repeatedly noted that, after forming an intention, they often became engaged in other ongoing tasks, which prevented them from keeping the original intention in focus. They acknowledged that without environmental cues (e.g., technological tools), they would not have been able to ensure the execution of the intention at a later point in time. Interestingly, students also admitted to other phenomena that we did not examine in our study, such as instances of false prospective memory retrieval (Retkoceri, 2021). For instance, students remembered to carry out an activity, but either at the wrong time or after becoming aware that it was not the right one. Sleep deprivation was another phenomenon that our study did not explore as related to prospective and retrospective memory failures. In agreement with the extant literature (Esposito et al., 2015; Goldberg et al., 2020), students often mentioned that they were aware that sleep deprivation made sustained attention harder and memory lapses more likely.

The present study has other limitations that need to be addressed in future research. First and foremost, memory abilities were not directly measured. Although robust evidence exists that metamemory reports are mostly accurate reflections of memory abilities (Kliegel and Jäger, 2006; Melo et al., 2021), evidence to the contrary (Arnold and Bayen, 2019) suggests caution in assuming accuracy. Thus, the extent to which metamemory and memory performance reflect one another is to be tested to determine its generalizability to the population selected for our study. Indeed, learners' ongoing assessment and monitoring of their learning may be inaccurate, leading to an unwarranted sense of competence (Soderstrom et al., 2015). Second, the selected student population entails students who are at the midpoint of their educational journey (sophomores and juniors). It is unclear whether freshmen who are closer in time to their verbatim educational experiences may display a stronger or weaker set of relationships between the variables of interest. Third, our study focuses on female undergraduate students within a society attempting to delete the scars of a past of patriarchy on women's sense of agency. The weak links between performance and self-efficacy beliefs and the null ones with metamemory measures may be traced to the difficulty these women experience in self-assessing their abilities in a changing society. In such a society, conflicts between old and new norms are commonplace. For instance, the promotion of gender equity and agency may permit adequate reporting of their abilities, whereas the patriarchal norms of the past may discourage it as a sign of lack of modesty.

In conclusion, within any educational setting, it is important that students accurately monitor and assess their learning and related abilities to ensure academic success. It is also important that instructors correctly understand their students' cognition and motivation to appropriately tailor instruction and develop interventions for learners at risk of failure. Our study is an attempt to understand the metamemory and self-efficacy beliefs of an understudied student population. It is just one of the steps we view as necessary for fostering the academic success of young women suddenly emerging from patriarchy.

## Data Availability

The raw data supporting the conclusions of this article will be made available by the authors, without undue reservation.

## Appendix

**Table A1:** Statements of the PRMQ and their categorical assignment (Crawford et al., 2003, p. 275).

	**Statement**	**P or R**
1	Do you decide to do something in a few minutes' time and then forget to do it?	P
2	Do you fail to recognize a place you have visited before?	R
3	Do you fail to do something you were supposed to do a few minutes later, even though it's there in front of you, like take a pill or turn off the kettle?	P
4	Do you forget something that you were told a few minutes before?	R
5	Do you forget appointments if you are not prompted by someone else or by a reminder such as a calendar or diary?	P
6	Do you fail to recognize a character in a radio or television show from scene to scene?	R
7	Do you forget to buy something you planned to buy, like a birthday card, even when you see the shop?	P
8	Do you fail to recall things that have happened to you in the last few days?	R
9	Do you repeat the same story to the same person on different occasions?	R
10	Do you intend to take something with you, before leaving a room or going out, but minutes later leave it behind, even though it's there in front of you?	P
11	Do you mislay something that you have just put down, like a magazine or glasses?	R
12	Do you fail to mention or give something to a visitor that you were asked to pass on?	P
13	Do you look at something without realizing you have seen it moments before?	R
14	If you tried to contact a friend or relative who was out, would you forget to try again later?	P
15	Do you forget what you watched on television the previous day?	R
16	Do you forget to tell someone something you had meant to mention a few minutes ago?	P

P or R, Prospective or retrospective.
